# Clinical features and risk factors of liver injury in patients with *Chlamydia psittaci* pneumonia- a retrospective analysis

**DOI:** 10.3389/fcimb.2023.1320758

**Published:** 2024-01-09

**Authors:** Xuejing Guo, Dan Zhu, Hui Chen

**Affiliations:** Department of Respiratory and Critical Care Medicine, Jinhua Municipal Central Hospital, Jinhua, China

**Keywords:** liver injury, *Chlamydia psittaci*, pneumonia, clinical features, lactate dehydrogenase

## Abstract

**Background:**

Research into the effects of *Chlamydia psittaci* pneumonia on the liver has emerged in the last few years. However, no studies have systematically described liver injury in patients with psittacosis. We present the first report on the clinical features and risk factors of liver injury in patients with *Chlamydia psittaci* pneumonia.

**Methods:**

We retrospectively collected the clinical parameters for 46 patients with *Chlamydia psittaci* pneumonia admitted to Jinhua Central Hospital from January 2019 to February 2023. We analyzed the liver function parameters and summarized the clinical characteristics and risk factors of liver injury.

**Results:**

Among the 46 patients, 39 (84.8%) had abnormal liver function, and 23 (50.0%) had liver injury. The ratio of patients with a history of alcohol consumption (39.1% vs. 4.3%, *P* =0.004) or severe pneumonia (56.5% vs. 26.1%, *P* =0.036) was higher in the liver injury group compared with the non-liver injury group. Laboratory tests showed higher lactate dehydrogenase (LDH) levels in the liver injury group (*P <*0.001). The optimal cut-off LDH level associated with liver injury was 473 IU/L as determined by ROC curve analysis. Furthermore, multivariate logistic regression analysis demonstrated that a history of alcohol consumption (odds ratio [OR] = 11.251; 95% confidence interval [CI] = 1.022 ~ 123.897, *P* =0.048) and an LDH level of ≥ 473IU/L (OR = 11.635, 95% CI = 1.832 ~ 73.869, *P* =0.009) were independent risk factors for liver injury.

**Conclusions:**

A history of alcohol consumption and an LDH level of over 473 IU/L are independent risk factors for *Chlamydia psittaci* pneumonia-related liver injury. It is recommended that particular attention be given to monitoring and evaluating liver function parameters when treating patients with *Chlamydia psittaci* pneumonia who have a high LDH level and history of alcohol consumption.

## Introduction

Psittacosis is a natural epidemic disease caused by the transmission of *Chlamydia psittaci* from poultry or other birds to humans, mainly through inhalation of pathogen-containing aerosols (Hogerwerf et al., 2020). Recent studies have demonstrated human-to-human transmission, suggesting that psittacosis should be evaluated as a high biosecurity risk and considered an emergent risk (Zhang et al., 2022b). Psittacosis is a systemic disease that can affect multiple systems and cause damage to multiple organs, with the lungs being the main target organ (Radomski et al., 2016). The clinical manifestations of *Chlamydia psittaci* pneumonia vary significantly, ranging from asymptomatic patients to patients with fever and respiratory symptoms, respiratory failure, severe pneumonia, or multi-organ failure (Meijer et al., 2021; Tang et al., 2023; Yang et al., 2023).

In recent years, reports of *Chlamydia psittaci* pneumonia have increased significantly. In addition to the lungs, the liver is one of the most commonly damaged organs, and *Chlamydia psittaci* pneumonia patients have been shown to exhibit varying levels of alanine aminotransferase (ALT) and aspartate aminotransferase (AST) (Yung and Grayson, 1988; Yang et al., 2022; Li et al., 2023). However, no studies have systematically described the clinical features of impaired liver function in these patients. In the current study, to provide evidence to inform the clinical management of and decision-making for patients with psittacosis, we retrospectively analyzed the liver function parameters of 46 patients with *Chlamydia psittaci* pneumonia hospitalized at Jinhua Central Hospital in China. Here, we present the first report of the clinical parameters and risk factors of impaired liver function in patients with *Chlamydia psittaci* pneumonia.

## Materials and methods

### Participants

This study included 46 patients who were diagnosed with *Chlamydia psittaci* pneumonia via metagenomic next-generation sequencing (mNGS) at Jinhua Central Hospital from January 2019 to February 2023. The inclusion criteria were as follows (1): patients who met the diagnostic criteria for community-acquired pneumonia and who were confirmed to be infected with *Chlamydia psittaci* according to mNGS of bronchoalveolar lavage fluid (BALF) or blood samples (2); patients with no other pathogens identified in blood culture, sputum culture, or BALF samples via direct slide analysis, cell count analysis, polymerase chain reaction testing for *Mycobacterium tuberculosis*, and fungal culture (3); patients with complete clinical information. Importantly, patients with a history of abnormal liver function were excluded. The Ethics Committee of Jinhua Central Hospital approved this study. All enrolled participants provided written informed consent. Basic information (including sex, age, epidemiological history, underlying medical conditions, body mass index (BMI), alcohol history, and occupation), observations regarding clinical manifestations, and laboratory test results were collected. The laboratory tests included routine blood testing, assessments of C-reactive protein (CRP), electrolytes, liver function, serum creatinine (SCr), lactate dehydrogenase (LDH), creatine kinase (CK), creatine kinase isoenzyme (CK-MB), and D-dimer and urinalysis.

### mNGS detection method

mNGS was carried out using the PDC-Seq™ system from DIAN Diagnostics (Hangzhou, China). This platform allows the detection of antimicrobial resistance genes. The detection process was as follows (1): A standardized process for collecting samples was performed. BALF samples were collected, and the potentially contaminated part of the front segment was discarded, while 10 ml of the remaining sample was placed into a sterile tube. Additionally, 3 to 5 mL blood samples were collected from patients, placed in a sample storage tube free of DNA and stored at 4°C. (2) Fully automated nucleic acid extraction was carried out with NGSmaster™. (3) Library construction was conducted using PCR-free technology. The Agilent 2100 Bioanalyzer Instrument and Qubit 3.0 (Thermo Fisher Scientific, USA) platform were used for library quality control. (4) An Illumina high-throughput sequencer was used for sequencing. (5) Pathogenic microbial gene data were analysis automatically using the provided software. High-quality sequencing data were obtained by removing low-quality and short (length <35 bp) reads.

### Diagnostic criteria

Abnormal liver function was defined as ALT >50 U/L, AST >40 U/L, gamma-glutamyl transferase (GGT) >60 U/L, alkaline phosphatase (ALP) >135 IU/L, and total bilirubin (TB) >25 umol/L. Liver injury was classified as hepatocellular-type, cholangiocyte-type or mixed-type injury. Patients were considered to have hepatocellular-type injury when their ALT or AST levels were over 3× the upper limit of normal (ULN), cholangiocyte-type injury when their ALP or GGT levels were over 2× the ULN, and mixed-type injury when their ALT or AST levels were over 3× the ULN and their ALP or GGT were over 2× the ULN (Cai et al., 2020). The diagnostic criteria for severe pneumonia were based on the “Guidelines for the Diagnosis and Treatment of Community-Acquired Pneumonia in Adults in China (2016 edition)” (Qu, 2016).

### Statistical analysis

SPSS 23.0 software was utilized to analyze all the data, and Graph Pad Prism 5.0 software was utilized to generate the figures. Normally distributed data (examined by the Kolmogorov-Smirnov test) were expressed as the mean ± SD or the median (interquartile range). Continuous variables according to a normal distribution were analyzed using the independent sample t-test or the Mann-Whitney U test. The optimal cut-off LDH level associated with liver injury was determined by receiver operating characteristic (ROC) curve analysis. The χ2 or Fisher exact tests were applied to compare categorical variables. The risk factors for liver injury in patients with *Chlamydia psittaci* pneumonia were explored by utilizing univariate and multivariate logistic regression models. All analyses used a two-sided test, and *P <*0.05 denoted a statistically significant difference.

## Results

### Clinical parameters

In all, 25 males and 21 females aged 40~81 (60.0 ± 9.9) years with *Chlamydia psittaci* pneumonia were included in this study. Of these patients, 32 had an explicit history of exposure to birds or poultry. There were 20 patients with background diseases, including 15 patients with hypertension, 5 with hepatitis B virus-related chronic liver disease (4 with hepatitis B infection, 1 with hepatitis B cirrhosis), and 2 with diabetes. Ten patients had a history of alcohol abuse and were drinking prior to the *Chlamydia psittaci* infection. The onset of disease occurred mainly in winter (22 patients, 47.8%). All patients had a high fever (maximum temperature ≥39°C), cough (30 patients), chest tightness (12 patients), headache and muscle aches (9 patients), gastrointestinal symptoms such as nausea, vomiting and diarrhea (8 patients), and delirium (4 patients). Thirty-three patients had abnormal breath sounds on chest auscultation, and 22 had pulmonary crackles. Nineteen patients manifested severe pneumonia, and 16 developed type I respiratory failure. Forty-one patients underwent antimicrobial resistance gene testing, but no associated genes were detected in any patient.

### Liver function related parameters

Among the enrolled patients with *Chlamydia psittaci* pneumonia, 39 (84.8%) had abnormal liver function, of which 65.2%, 80.4%, 30.4%, and 39.1% had elevated ALT, AST, ALP, and GGT levels, respectively. Twenty-three patients (50.0%) had liver injury of which 15.2%, 39.1%, 8.7%, and 23.9% had significantly elevated ALT, AST, ALP, and GGT levels, respectively. Eleven patients had hepatocellular-type injury, 4 had cholangiocyte-type injury, and 8 had mixed-type injury, indicating that the primary type was hepatocellular injury. The specific liver function parameters are described in **Table 1**. In addition, it is worth mentioning that 5 patients with hepatitis B virus-related chronic liver disease all developed abnormal liver function, and 2 of them met the diagnostic criteria for liver injury.

**Table 1:** Liver function parameters of patients with *Chlamydia psittaci* pneumonia.

Characteristic	Mean	SD	Median	Proportion of abnormal liver function (%)	Proportion of liver injury (%)
ALT (IU/L)	89.8	73.2	70.4	30 (65.2)	7 (15.2)
AST (IU/L)	158.4	163	87.2	37 (80.4)	18 (39.1)
ALP (IU/L)	147.4	109.7	110	14 (30.4)	4 (8.7)
GGT (U/L)	92	88.2	59.8	18 (39.1)	11 (23.9)
TB (umol/L)	19	15.1	12.5	8 (17.4)	/

/, Not available.

### Analysis of clinical parameters

The clinical parameters of patients with *Chlamydial psittaci* pneumonia are shown in **Table 2**. In the liver injury group, 9 patients had consumed alcohol previously, which was dramatically higher than the number in the non-liver injury group (*P* =0.004). In addition, 13 patients in the liver injury group developed severe pneumonia, while only 6 patients did in the non-liver injury group (*P* =0.036). Compared with that in the non-liver injury group, the proportion of patients with respiratory failure was higher in the liver injury group, but the difference was not statistically significant (*P* =0.063). No significant differences were observed in sex, age, background diseases, hepatitis B infection status, cirrhosis, BMI, or clinical symptoms (such as cough, chest tightness, headache, muscle aches, vomiting, and diarrhea). Additionally, 15 patients (65.2%) in the liver injury group had a contact history with birds or poultry (**Supplementary Table 1**).

**Table 2:** Clinical features of liver injury in patients with *Chlamydia psittaci* pneumonia.

Clinical Feature	Cases	Liver injury	Non-liver injury	*P* value
	N=46	N=23	N=23	
Male	25	15	10	0.139
Age≥60	23	12	11	0.768
Background disease	20	8	12	0.234
Hepatitis B,/cirrhosis	5	2	3	0.636
History of alcohol consumption	10	9	1	0.004
BMI≥24	15	7	8	0.753
Cough	30	14	16	0.536
Chest tightness	12	5	7	0.502
Headache, muscle aches	9	5	4	1
Vomiting diarrhea	8	4	4	1
Severe pneumonia	19	13	6	0.036
Respiratory failure	16	11	5	0.063

### Analysis of the laboratory test results

**Figure 1:**
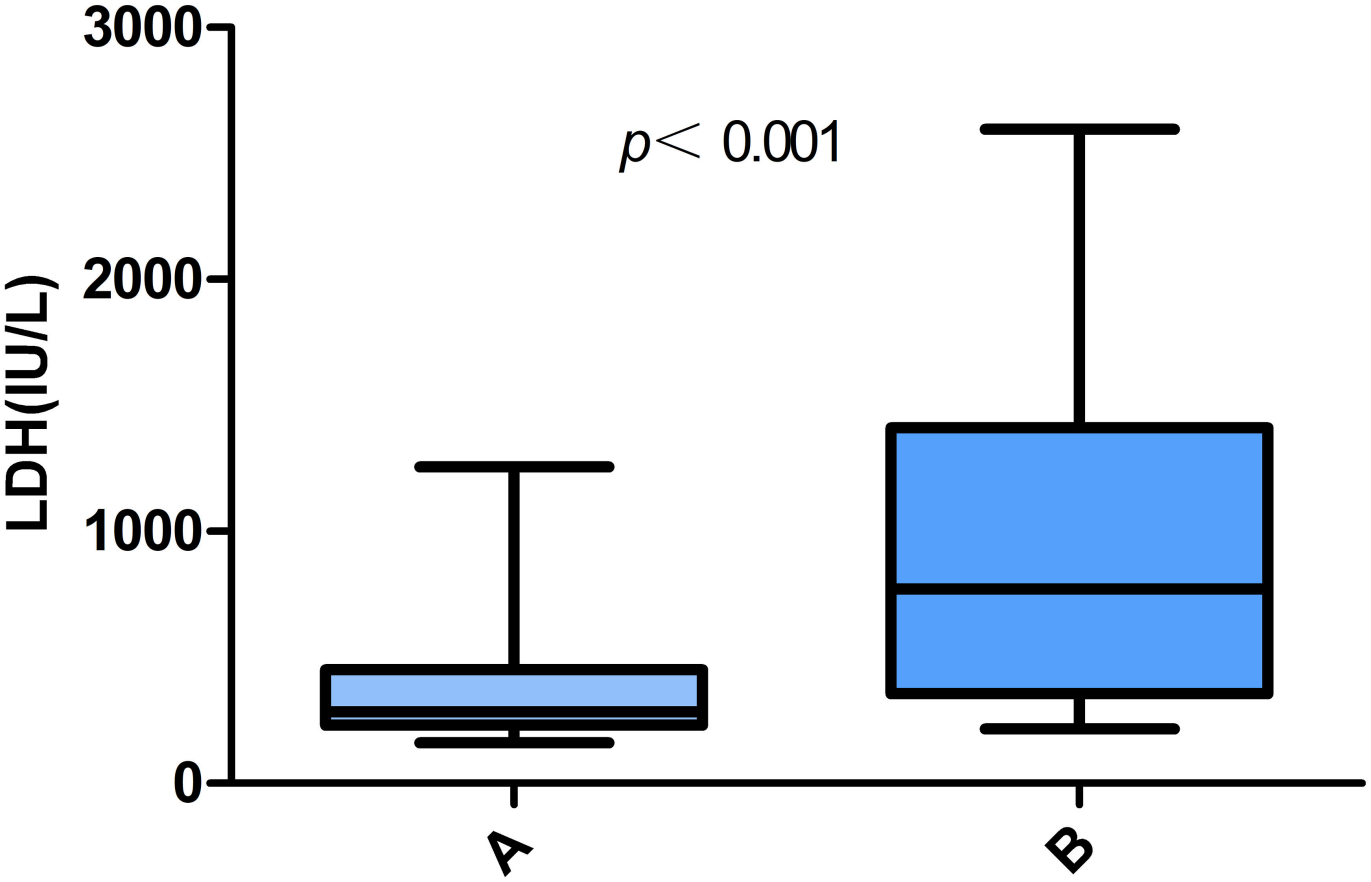
Comparative analysis of LDH levels in patients with *Chlamydia psittaci* pneumonia. **(A)** Non-liver injury; **(B)** liver injury.

**Figure 2:**
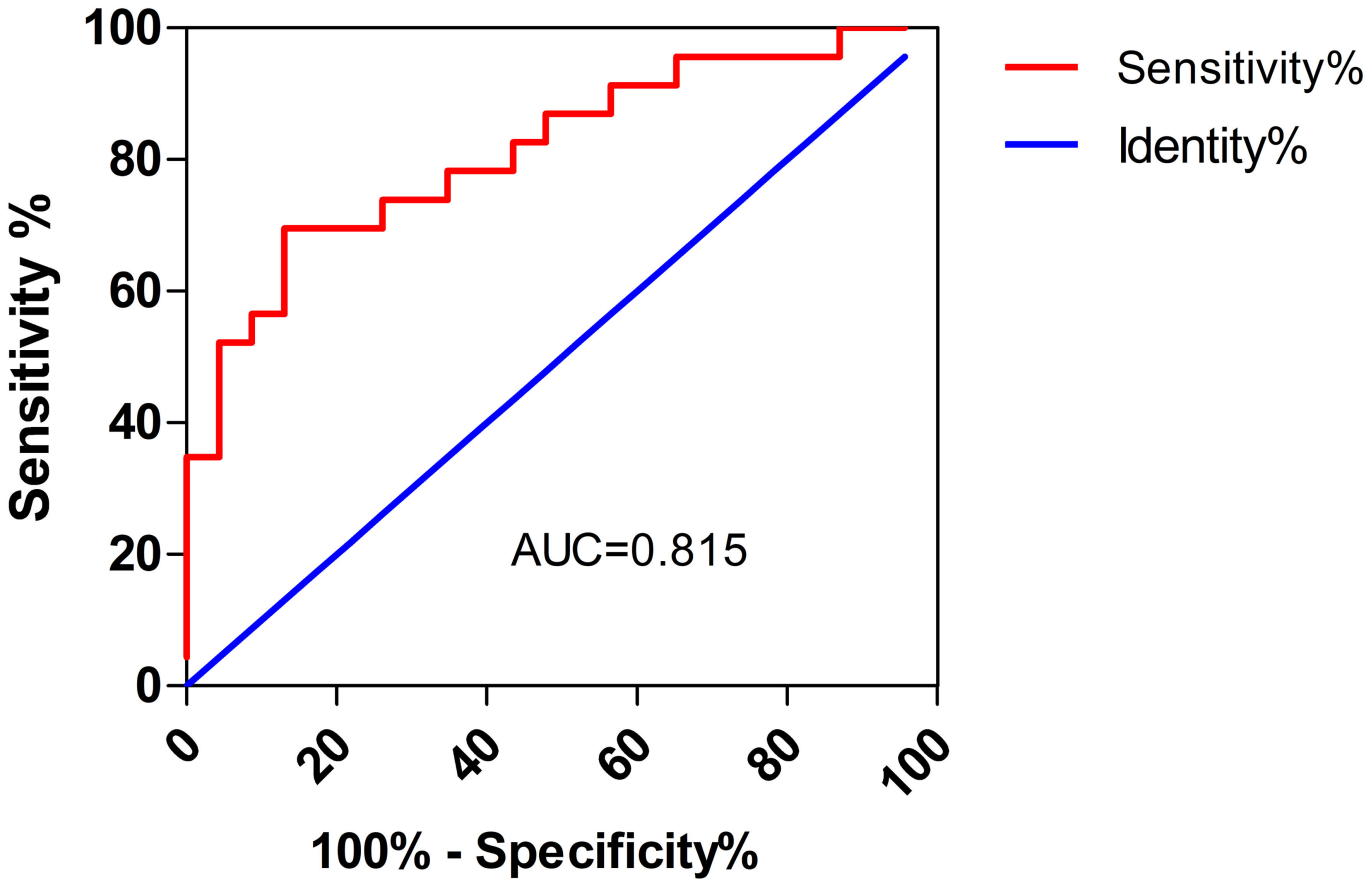
ROC curve analysis of LDH levels and liver injury.

The laboratory test results for patients with *Chlamydia psittaci* pneumonia are shown in **Table 3**. Most patients with *Chlamydia psittaci* pneumonia had a normal white blood cell count, lymphopenia, and varying degrees of elevated CRP and D-dimer levels. Electrolyte metabolism disturbances were common; hypokalemia was observed in 25 patients, and hyponatremia was found in 34 patients. In addition, urine protein-positive and urinary occult blood-positive results were found in 50.0% and 67.4% of patients, respectively, suggesting that these are common results in patients with *Chlamydia psittaci* pneumonia. In the group of patients with liver injury, some presented elevated troponin I (6 out of 17 patients; 35.3%) and elevated brain natriuretic peptide (BNP) levels (10 out of 17 patients; 58.8%). Notably, patients in the liver injury group had significantly higher LDH levels than those in the non-liver injury group (*P <*0.001) (**Figure 1**). The optimal cut-off LDH level associated with liver injury was 473 IU/L with a sensitivity of 0.696 and a specificity of 0.870 according to ROC curve analysis. The AUC was 0.815, and the 95% CI was 0.691-0.938 (*P <*0.001) (**Figure 2**). However, the differences in the results for routine blood testing, CRP, SCr, CK, CK-MB, D-dimer, electrolyte metabolism disorders, urine protein-positive proportion, and urinary occult blood-positive ratio between the two groups were not significant.

**Table 3:** Analysis of laboratory test results for *Chlamydia psittaci* pneumonia patients with liver injury.

Laboratory test	Cases	Liver injury N=23	Non-liver injury N=23	*P* value
	N=46			
WBC (*10 (Yung and Grayson, 1988)*10^9^/L)	6.3 (5.2,8,9)	5.7 (4.8,8.8)	7.8 (5.3,10.8)	0.317
N (*10 (Yung and Grayson, 1988)*10^9^/L)	5.6 (4.1,8.3)	5.4 (4.4,8.2)	7.0 (3.9,10.0)	0.692
L (*10 (Yung and Grayson, 1988)*10^9^/L)	0.53 ± 0.29	0.48 ± 0.30	0.58 ± 0.27	0.23
PLT (*10 (Yung and Grayson, 1988)*10^9^/L)	161 (96,224)	106 (59,222)	170 (141,235)	0.097
CRP (mg/L)	143 (88,174)	168 (136,187)	140 (98,166)	0.184
SCr (umol/L)	68.4 (58.2,88,8)	68.8 (58.3,99.0)	71.6 (60.5,87.8)	0.568
LDH (IU/L)	410 (240,867)	898 (568,1411)	286 (232,451)	<0.001
CK (IU/L)	100 (43,262)	171 (75,946)	89 (46,200)	0.356
CK-MB (IU/L)	9.0 (6.3,15.2)	10.6 (5.0,20.4)	8.0 (6.1,16.0)	0.947
D-dimer (ug/L)	1539 (769,3862)	1910 (1451, 5822)	973 (702,1956)	0.063
Hypokalemia (%)	25 (54.3)	15 (65.2)	10 (43.5)	0.139
Hyponatremia (%)	34 (73.9)	20 (87.0)	14 (60.9)	0.091
Hypochloremia (%)	15 (32.6)	10 (43.5)	5 (21.7)	0.116
Urine protein positive (%)	23 (50.0)	12 (52.2)	11 (47.8)	0.768
Urine occult blood positive (%)	31 (67.4)	15 (65.2)	16 (69.6)	1

Normal range: WBC white blood cells (3.5-9.5*10^9^/L); N neutrophils (1.8-6.3* 10^9^/L); L lymphocytes (1.1-3.2*10^9^/L); PLT platelets (125-350*10^9^/L); CRP C-reactive protein (<8 mg/L); SCr serum creatinine (40-135 umol/L); LDH lactate dehydrogenase (120-246 IU/L); CK creatine kinase (55-170IU/L); CK-MB creatine kinase isoenzyme (0-16IU/L); D-dimer (0-500ug/L); potassium (3.5-5.3mmol/l); sodium (137-145mmol/l); chlorine (98-107mmol/L).

### Risk factors for abnormal liver function

Multivariate analysis showed that an elevated LDH level and a history of alcohol consumption significantly increased the risk of liver injury in patients with *Chlamydia psittaci* pneumonia (OR=11.635, 95% CI=1.832-73.869, *P*=0.009; OR=11.251, 95% CI=1.022-123.897, *P*=0.048) (**Table 4**).

**Table 4:** Logistic regression analysis of liver injury in patients with *Chlamydia psittaci* pneumonia.

Clinical feature		OR	95% CI	p-value
LDH group (IU/L)	≥473vs<473	11.635	1.832-73.869	0.009
History of alcohol consumption	Yes vs. No	11.251	1.022-123.897	0.048
Severe pneumonia	Yes vs. No	1.242	0.200-7.729	0.815

## Discussion

To our knowledge, this is likely the first and most comprehensive study describing the results of liver function tests in patients with *Chlamydia psittaci* pneumonia. In the current study, 84.8% of patients with *Chlamydia psittaci* pneumonia developed abnormal liver function at initial diagnosis, and 50% developed liver injury. Our results confirmed that a history of alcohol consumption and an LDH level over 473 IU/L were positively associated with the elevated risk of liver injury.

One meta-analysis suggested that the incidence of *Chlamydia psittaci* pneumonia was approximately 1% (Hogerwerf et al., 2017). Testing for *Chlamydia psittaci* is usually not included in routine microbiological diagnosis. Therefore, the incidence and disease burden of human psittacosis may be significantly underestimated. After inhalation, *Chlamydia psittaci*, a gram-negative intracellular obligate bacterium, first proliferates in the mononuclear-macrophage cells of the liver and spleen and then spreads to the lungs and other organs through the blood circulation (Shen et al., 2021), which partially explains why liver dysfunction is common in patients with *Chlamydia psittaci* pneumonia.

This study showed that most patients (69.6%) had a definite bird or poultry exposure history. The clinical symptoms of these patients were not identical, and the typical symptoms were high fever; additionally, some patients presented with cough, headache, muscle aches, nausea, vomiting, diarrhea, and delirium. Our results illustrated that the incidence of abnormal liver function and liver injury in patients with *Chlamydia psittaci* pneumonia who were admitted to our center was high, namely, 84.8% of patients with *Chlamydia psittaci* pneumonia had higher levels of ALT, AST, ALP, GGT, and TB at initial diagnosis, among which an elevated AST level was the most common. Similar to previous results, an elevated AST level was more common than an elevated ALT level (Su et al., 2021; Li et al., 2022; Yin et al., 2022). A retrospective study including 52 patients with *Chlamydia psittaci* pneumonia conducted by Li et al. showed that the elevation ratios in AST and ALT were 86.5% and 75.0% (Li et al., 2022), respectively. Branley et al. found that 60% (24 of 40) of patients showed abnormal liver function upon admission and aminotransferase elevation within 2× the ULN (Branley et al., 2014). However, most of the previously reported studies only summarized the results of liver enzymes without systematically analyzing other liver function parameters such as the levels of ALP, GGT, and TB. In the current study, 50% of patients developed liver injury, and the results showed that *Chlamydia psittaci* mainly caused hepatocellular liver injury, with the AST level being a relatively sensitive indicator.

This analysis summarized the clinical features and laboratory parameters of liver injury and non-liver injury in patients with *Chlamydia psittaci* pneumonia and found that liver injury was more common in patients with a history of alcohol consumption, severe pneumonia, and elevated LDH levels. In 2022, a retrospective study analyzed the clinical features of 30 patients with *Chlamydia psittaci* pneumonia and demonstrated that the ratio of patients with abnormal liver function was significantly higher in the severe group (*P* < 0.001) (Liang et al., 2022). Su et al. reported similar results that patients with severe *Chlamydia psittaci* pneumonia showed higher levels of AST and LDH (Su et al., 2021). In addition, our data suggested that liver injury was not associated with background hepatitis B virus infection, consistent with a recent finding reported by Tang et al. (Tang et al., 2022). However, all 5 patients with hepatitis B virus-related chronic liver disease had abnormal liver function, and hepatitis B virus infection may be a predisposing factor, which needs to be confirmed by more studies. We found that most patients with psittacosis had normal WBC counts, lymphopenia, and elevated levels of CRP, D-dimer, and LDH, consistent with previously published studies (Ni et al., 2023; Wu et al., 2023). Further regression analysis showed that a history of alcohol consumption and an LDH level over 473 IU/L could be independent risk factors for liver injury in patients with psittacosis.

Previous studies have partly revealed the possible mechanisms by which *Chlamydia psittaci* causes liver injury. Chlamydial species reside primarily within peripheral blood mononuclear cells in different organs including the liver, which may contribute to amplify the local and systemic inflammatory response against chlamydial infection (Contini et al., 2011). *Chlamydia psittaci* shows similar biological behavior, which explains the liver injury in infected patients to some extent. *Chlamydia psittaci* can be effectively transmitted to patients through rapid entry into a wide range of host cells through specific surface proteins located in mitochondria and the Golgi apparatus via intracellular transport pathways; the expression of immunological genes is upregulated in response to *Chlamydia psittaci* infection, resulting in fulminant systemic disease (Knittler and Sachse, 2015). When the pathogen invades the body, multiple inflammatory factors (such as IL-6, TNF-a, and IL-1RN) are upregulated, thereby enhancing the inflammatory response, promoting abnormal liver function, and producing CRP and D-dimer (Zhang et al., 2022a). In addition, assessment of liver biopsy samples from patients with *Chlamydia psittaci*-induced liver injury show granulomas with Kupffer cell hyperplasia (Carella et al., 1996), suggesting to some extent that inflammatory cell infiltration with the release of multiple inflammatory factors is closely related to the occurrence and development of liver injury.

Liver injury in patients with psittacosis implies severe hepatic insufficiency, which requires frequent liver function monitoring and careful antibiotic evaluation, which limits the use of quinolones, macrolides, and traditional tetracyclines. Studies have shown that omacycline cannot be metabolized in the body and is mainly excreted through feces, which is safe for patients with hepatic insufficiency (Rodvold et al., 2020). The effectiveness of doxycycline in patients with *Chlamydia psittaci* infection has also been demonstrated (Fang et al., 2022). Overall, doxycycline is a suitable choice for patients with psittaci-related liver injury.

This study had several limitations that must be noted. First, it was a retrospective study conducted at a single medical center, and only a few participants were enrolled. In the future, a multicenter, large-scale cohort study is necessary to further confirm the abnormal liver function and liver injury in Chinese patients with *Chlamydia psittaci* pneumonia. Second, data on liver injury caused by other factors, such as Chinese herbal medicines and self-medicated antipyretic drugs, before admittance to our hospital were unavailable. We excluded participants with previous abnormal liver function treated at our research center, and liver function tests were performed for all the participants before taking drugs at admission, which excluded the possibility of drug-induced liver damage to a large extent. Therefore, the liver injury of patients should be caused by *Chlamydia psittaci* itself.

In conclusion, the current study evaluated the clinical features of patients with *Chlamydia psittaci* pneumonia and abnormal liver function. A history of alcohol consumption and an LDH level over 473 IU/L could serve as independent risk factors for liver injury in patients with psittacosis, and these patients require close clinical observation and medical evaluation.

## Data availability statement

The data presented in the study are deposited in the SRA repository, accession number SRP480695.

## Ethics statement

The studies involving humans were approved by Jinhua Central Hospital Medical Ethics Review Committee, Jinhua Central Hospital. The studies were conducted in accordance with the local legislation and institutional requirements. The human samples used in this study were acquired from a by- product of routine care or industry. Written informed consent for participation was not required from the participants or the participants’ legal guardians/next of kin in accordance with the national legislation and institutional requirements. Written informed consent was obtained from the individual(s) for the publication of any potentially identifiable images or data included in this article.

## Author contributions

XG: Writing – original draft. DZ: Writing – review & editing. HC: Writing – review & editing.
