# Supplementary material for: Clinical features and risk factors of liver injury in patients with Chlamydia psittaci pneumonia- a retrospective analysis
Source: Front Cell Infect Microbiol. 2024 Jan 9;13:1320758. doi: 10.3389/fcimb.2023.1320758 (PMC10803654; doi:10.3389/fcimb.2023.1320758)
Supplement: Supplementary file 1 [file Table_1.docx]

Supplementary Table1 Clinical data for patients in the liver injury group with birds or poultry exposure

| Case | Age | Sex | | Contact history | | Exposure time | |
| --- | --- | --- | --- | --- | --- | --- | --- |
| 1  2  3  4  5  6  7  8  9  10  11  12  13  14  15 | 60  55  58  64  55  64  65  62  55  73  67  52  70  71  49 | Male  Male  Male  Male  Male  Male  Female  Male  Male  Female  Male  Male  Male  Male  Male | Poultry raising  Poultry butcher  Birds contact  Birds contact  Poultry raising  Poultry raising  Poultry raising  Poultry raising  Poultry butcher  Poultry raising  Birds contact  Poultry raising  Poultry raising  Poultry raising  Poultry butcher | | Long-term  2 months  4 months  1 week  2 months  1 month  Long-term  Long-term  2 days  Long-term  2 months  2 weeks  Long-term  Long-term  1 week | |  |
